# The Use of Supervised Learning Models in Studying Agonistic Behavior and Communication in Weakly Electric Fish

**DOI:** 10.3389/fnbeh.2021.718491

**Published:** 2021-10-11

**Authors:** Federico Pedraja, Hendrik Herzog, Jacob Engelmann, Sarah Nicola Jung

**Affiliations:** ^1^Department of Neuroscience, Zuckerman Mind Brain Behavior Institute, Columbia University, New York, NY, United States; ^2^Department of Neuroethology/Sensory Ecology, Institute for Zoology, University of Bonn, Bonn, Germany; ^3^Active Sensing, Faculty of Biology, Bielefeld University, Bielefeld, Germany

**Keywords:** supervised learning, agonistic behavior, weakly electric fish, passive electric image, active electric image

## Abstract

Despite considerable advances, studying electrocommunication of weakly electric fish, particularly in pulse-type species, is challenging as very short signal epochs at variable intervals from a few hertz up to more than 100 Hz need to be assigned to individuals. In this study, we show that supervised learning approaches offer a promising tool to automate or semiautomate the workflow, and thereby allowing the analysis of much longer episodes of behavior in a reasonable amount of time. We provide a detailed workflow mainly based on open resource software. We demonstrate the usefulness by applying the approach to the analysis of dyadic interactions of *Gnathonemus petersii*. Coupling of the proposed methods with a boundary element modeling approach, we are thereby able to model the information gained and provided during agonistic encounters. The data indicate that the passive electrosensory input, in particular, provides sufficient information to localize a contender during the pre-contest phase, fish did not use or rely on the theoretically also available sensory information of the contest outcome-determining size difference between contenders before engaging in agonistic behavior.

## Introduction

Weakly electric fish are active at night and are frequently found in dark and turbid environments (Moller et al., 1979). The specialized electric sense enables weakly electric fish well adapted to cope with the specific challenges imposed by this lifestyle (Carlson and Sisneros, 2019). They produce electric signals for both, electrolocalization and electrocommunication with conspecifics (Möhres, 1957; Lissmann, 1958; Lissmann and Machin, 1958; Moller, 1970). These signals are generated through an electric organ (EO) distributed along the trunk (South American weakly electric fish) or the tail (African weakly electric fish). The synchronous discharge of these organs [electric organ discharge (EOD)] is used to emit either intermittent or continuous electric fields (Gallant, 2019). EODs are low in amplitude (often in the order of 1 mV) and, in addition, these signals attenuate steeply with distance from the emitting fish (Rasnow, 1996; Sicardi et al., 2000; Chen et al., 2005; Nelson and MacIver, 2006).

With respect to electrolocation, it is known that weakly electric fish electrically locate objects in the dark and even discriminate between objects relying on various object features (von der Emde, 1990, 1999; von der Emde and Bleckmann, 1998; Schwarz and von der Emde, 2000). Furthermore, it has been investigated that electric fish also use their electric sense for spatial navigation (Jun et al., 2016; Jung et al., 2019). The role of EODs in electrocommunication has also been studied although these studies until recently were constrained to lab conditions (Moller, 1970; Walz et al., 2013). Recent (costly) advances have made it possible to now also study electric communication (of wave-type species) in the wild (Henninger et al., 2018, 2020; Madhav et al., 2018; Raab et al., 2021).

Weakly electric fish are either a wave type, i.e., emitting EODs continuously, or a pulse type, i.e., emitting short EOD pulses with variable intervals between pulses. Wave-type weakly electric fish emit with relatively stable EOD frequencies (Walz et al., 2013; Henninger et al., 2020) and this continuous discharge at stable and individual-specific frequencies makes the attribution to individuals comparatively easy (Madhav et al., 2018). In pulse-type electric fish, however, the inter-discharge time is typically larger than the EOD duration, making a frequency-based assignment of EODs impossible. Furthermore, the EODs of pulse-type species typically show relatively small intrasexual individual differences (Carlson and Arnegard, 2011; Krahe, 2019). In *Gnathonemus petersii*, the role of androgens and estrogen on the EOD waveform has been demonstrated (Landsman et al., 1990). In captivity, without any hormonal treatment, interindividual EOD differences are rather small. This holds specifically true for individuals of the same sex (Landsman et al., 1990; Landsman, 1993). The study of electrocommunication in pulse-type species is still limited by the laborious manual analysis of very short behavioral sequences (Moller and Bauert, 1973; Gebhardt et al., 2012). A successful workaround has been the use of artificial fish that emit pre-defined EOD sequences. This has proven as a successful means to study the impact of the defined EOD sequences on the behavior groups of fish and individuals (Donati et al., 2016; Pannhausen et al., 2018; Worm et al., 2018).

Our study aims to provide tools to facilitate the assignment of EOD in pulse-type fish. While we establish a workflow for the interaction of dyads, the approach in principle can be scaled up to larger groups. In contrast to the aforementioned techniques that allow purely electrical tracking and identification of individuals, the workflow established in this study aims to facilitate offline and lab-oriented work and requires the extraction of fish locations using common visual tracking methods.

In brief, our approach employs supervised learning methods to first track fish individuals and then use the position data to assign EODs to the individuals. The position tracking is based on open-source software (https://sleap.ai/, version: 1.016) that is used for estimating the positions of animal body parts (Pereira et al., 2019, 2020). It supports multianimal pose estimation and tracking and includes an advanced labeling/training graphical user interface (GUI) for active learning and proofreading. Implementing a decision-tree model that was trained with prerecorded data, we then used the position data to attribute EODs to individuals. The model is part of the Scikit-learn package, implemented in Python, and also freely available (Pedregosa et al., 2011). The combination of visual tracking and supervised learning resulted in high performances and accuracy of position estimation and the attribution of EODs to individuals. Error rates were below 5% and could be reduced further with a small to an intermediate effort by using the interactive social leap estimates animal poses (SLEAP) GUI and resorting to the manual assignment of EODs in a small and automatically identified subset of the data.

To demonstrate the suitability of the proposed workflow, we applied it to study the aggressive behavior in *G. petersii* during dyadic interactions. These fish are territorial and often live in fixed groups with social ranking. In residence-intruder interactions, the aggression of the resident fish toward an intruder has been described (Crockett, 1986). The outcome of such encounters can depend on the body size, meaning that the larger fish has a higher probability to win the fight (Bell et al., 1974; Terleph, 2004). As agonistic behavior can be costly, we now ask if the electric sense contributes to a precontest assessment of the quality of a potential contender before engaging in agonistic interactions. The resource holding potential (RHP), i.e., the ability to win a possible fight, is frequently assessed based on far-ranging sensory input (Nelson, 2006), but these are unavailable or unreliable during the nocturnal encounters of weakly electric fish. Therefore, we ask if *G. petersii* can evaluate the outcome of a fight before the first physical contact: We hypothesized that fish would not initiate an aggressive contact if they can determine beforehand that they are likely to lose. If so, the electrosensory information may either be passive and/or active. The former modality allows the perception of external electric fields created by inanimate or living organisms, including the EODs of other weakly electric fish. In contrast, the active electrosensory modality relies on the perceptions of the self-generated electric field. Here, the modulation of this field through nearby objects having a different conductivity or capacity from the surrounding water provides environmental information used by these fish to reconstruct their surrounding (Knudsen, 1975; von der Emde, 1999). A previous study on the South American weakly electric fish *Gymnotus omarorum* tested the RHP of the contender through modeling of the sensory input (Pedraja et al., 2016). We do a follow up on this study using an evolutionary distinct weakly electric fish lineage to demonstrate the power of supervised learning methods for research on weakly electric fish communication. Passive and active electroreception is mediated through different electroreceptors that both occur and are distributed over the animal skin. The electric pattern, i.e., the distribution of local field intensities, provides the relevant input to both modalities. This spatial distribution is referred to as the electric image (EI) (Caputi and Budelli, 2006). To distinguish between active and passive sources, we will refer to the sensory images generated by the presence of external electric fields as passive EI and to those images generated by the distortions through elements in the electrosensory scenery as active EI.

## Materials and Methods

### Setup

The experimental tank had a size of 66 × 72 cm and was filled up to a water level of 12 cm. Seven pairs of electrodes were mounted on the tank walls. Electrodes were made of coated thin silver wires attached to plastic rods, the tips of the wires were exposed for about 1–2 mm and placed 5 cm above the tank floor. The floor of the tank was filled with small glass beads to allow illumination from below.

Electric organ discharges were recorded with an Axon Instruments amplifier (Foster City, CA, USA; Cyber Amp 380), digitized at 125 kHz (National Instrument, Austin, TX, USA; PCI-6251 M, 24 bit). Amplification and filter settings (high-pass 300 Hz and low-pass 10 kHZ) of the amplifier were controlled through the software (National Instruments, Austin, TX, CyberControl, 1.1.0.12).

IR-LEDs (880 nm) were placed below the tank to illuminate the tank from below. *G. petersii* has been reported to not perceiving IR light of this wavelength (Ciali et al., 1997). Videos of fish were captured from above (Mako 130B mono, AVT imaging, frame rate: 30 or 10 Hz, stored in an AVI format). Each exposure also triggered a transistor-transistor logic (TTL) pulse that was recorded alongside the EOD data (125 kHz sampling rate, 12 bit, audio card). These pulses were used to synchronize video and EOD data. EOD and video recordings were initiated through custom written MATLAB scripts (32 bit; R2013, MathWorks, Natick, MA, USA).

### Animals

Fish (*n* = 6) were housed in a large tank with partitions to keep individuals from physical interactions outside the experimental time. The light/dark cycle was 12/12 h, and all experiments were conducted during the subjective night time of the fish. Water conductivity in the experimental tanks was 120 ± 5 and 160 ± 20 μS/cm in the holding tanks. Water temperature was regulated to 22 ± 2°C by heating the experimental room to avoid electric noise through aquarium heaters.

### Data Sets

Two different data sets were acquired. *Data set I* is based on two fish of equal size, where we obtained 20 60 s recordings of each of these fish exploring the tank individually. This data set was used to train supervised learning models such as the random forest regressor (RFR) model and multilayer perceptron (MLP) models (see section Types of Supervised Learning Models for Regression). To estimate the performance of EOD allocation, we also recorded and analyzed 10 60-s long videos with both fish interacting in the tank. The performance of EOD allocation was analyzed by contrasting this automated allocation to a human-observer-based allocation of EODs (see section Using Supervised Learning to Predict the Fish Position of Real Fish).

With *data set II*, we then further evaluate the suitability of the workflow by addressing aggressive behavior during dyadic interactions of fish of different sizes (*n* = 6). Size difference and thus the differences in EOD amplitude could in theory influence the accuracy of the EOD allocation, thus this data set also served to test for the robustness of the used model. Our behavioral analysis focuses on the first approach between two fish. With the 6 individuals, we could have tested 15 possible pairings. However, we excluded the interaction of the two fish used in the first data set to make sure that fish had not a chance to have *a priori* knowledge about their contender. Of the remaining 14 pairings, we needed to exclude 1 pairing, where fish approached each other swimming backward making it impossible to determine who initiated the contact.

### Two-Dimensional Representation of Electric Potentials

Python (Version 3.7) was used to simulate the potential at the electrodes in the horizontal plane of the experimental setup. For this, virtual electric dipoles (virtual fish) were randomly positioned and oriented in this plane (2,500 positions tested). To minimize border effects, virtual fish were at least 10 cm from the walls of the tank. In total, 20 virtual fish of 8–15 cm length were simulated. Thus, the used data set contained the variables fish length, *x* and *y* coordinates of the fish center, and the sine and cosine of the angle, the virtual fish was oriented at and the seven potentials of the electrodes.

The electric field of the virtual fish was modeled based on a simple dipole, i.e., point charges of equal amplitude and opposite polarity located in the tail and the head of the animals, respectively. The potential at the electrodes was calculated using the equation for an electric dipole potential:


(1) $$\begin{array}{l} \text{V}={\text{k}}^{*}{\text{q}}^{*}\left(1/\text{R}1-1/\text{R}2\right) \end{array}$$

where V is the resulting voltage, k is the Boltzmann constant, and R1 and R2 are the distances of the head and tail position to the electrodes of interest, respectively.

We were only interested in the relative differences between electrode pairs. Therefore, charge *q* was set to 1/*k* throughout all simulations, this resulted in electrode voltages within the range of ±1 V irrespective of the position of the fish in the tank. For the electrodes, we simulated both differential and single-ended recordings against the ground. Differential recordings were simulated as the potential difference of electrode pairs, whereas single-ended recordings were simulated with the reference potential in the center of the tank.

### Types of Supervised Learning Models for Regression

**Figure 1:**
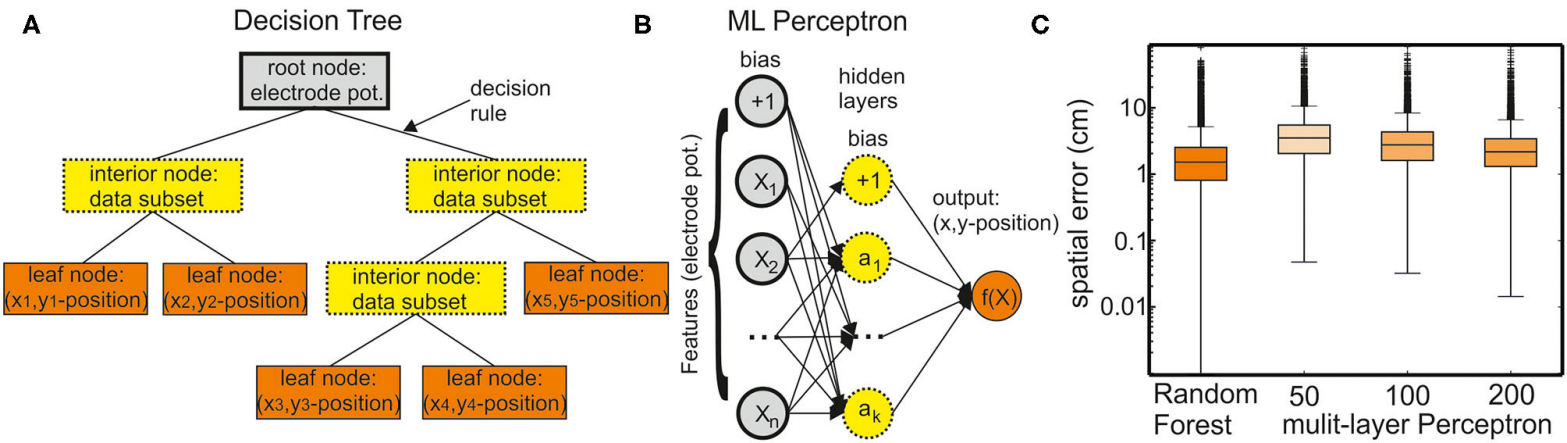
Supervised learning methods for regression. **(A)** A decision tree comprises a root node containing all input data features, interior nodes containing subsets of the data, and leaf nodes containing the final target values. A decision tree can have different numbers of interior nodes and leaf nodes. The goal of a decision tree is to create a model that predicts the value of a target variable (*x* and *y* position of the six fish skeleton coordinates) by learning the decision rules derived from the data (electrode potentials). **(B)** A multilayer perceptron (MLP) model can learn a nonlinear function approximation from a set of features and a target. The input layer comprises a set of neurons representing these input features (electrode potentials). Each neuron in the hidden layer transforms the values from the previous layer through a weighted linear summation followed by a nonlinear activation function. However, in case of a regressor model, the activation function is omitted (set to the identity function). The output layer receives the values from the last hidden layer and transforms them into output values (*x* and *y* positions of the fish skeleton). **(C)** Comparison of the regression performance of the random forest regressor (RFR) and MLP models with different numbers of hidden layers. The spatial error is defined as the average distance between true and the model-estimated head position (anchor point, see also Figure 2).

We tested several types of supervised learning models to predict fish position and orientation based on the electric potentials. We now describe the two models that we determined as suitable from pretests. To compare their performance, we used the estimation errors of the models when allocating EODs for real fish (data set I) (Figure 1).

#### Random Forests Regressor

A nonparametric supervised learning method, decision trees, is used for classification and regression (Figure 1A). The objective of a decision tree is to establish a model for predicting the value of a target from the input data features through learning simple decision rules. Each decision tree has branches and three types of nodes: the root node is the initial node, which represents the entire sample (in this case electrode potentials). The interior nodes represent the features of a data set, and the branches represent the decision rules. Finally, the leaf nodes represent the outcome (in this case *x* and *y* positions of the fish skeleton). Decision-tree models are prone to overfitting. Random forests are an ensemble learning method for classification, regression, and the other tasks that operate by constructing a multitude of decision trees at training time. They provide a solution to the problem of overfitting. In random forests, each tree in the ensemble is built from a sample drawn with replacement (i.e., a bootstrap sample) from the training set. For random forest regression tasks, the average/median/most common vote prediction of the individual trees is returned.

#### Multilayer Perceptron Model for Regression

This type of model can learn a function approximation from a set of features and the target (Figure 1B). The input layer comprises a set of neurons representing the input features (in this case: electrode potentials). Each neuron in the hidden layer transforms the values from the previous layer with a weighted linear summation. In a classification model, this is followed by a nonlinear activation function. However, in case of regression, the activation function is set to an identity function. The output layer receives the values from the last hidden layer and transforms them into output values (in this case: *x* and *y* positions of the fish skeleton).

### Using Supervised Learning to Predict Fish Position and Orientation of Simulated Data

A supervised learning algorithm was applied to predict fish position and orientation based on the simulated potentials. Specifically, we used the RFR model from the Scikit-learn package (Version 0.22.2) with default settings and the number of trees in the forest set to 25 (Pedregosa et al., 2011). The electrode potentials to the model were provided as independent variables from which the location, orientation, and size of the fish had to be predicted. A grid search optimization (“GridSearchCV” from the Scikit-learn package) was used with 25 iterations to tune the RFR. To evaluate the learning, the data set was split into a training (75% of the data) and a test set (25% of the data) that was not included in the learning phase. The supervised learning success was verified by a built-in metric (“score” function in the Scikit-learn package). The score function returns the coefficient *R*^2^ that is defined as (1-*u*/*v*), where *u* is the residual sum of squares: sum [(true position–predicted position)^2^], and *v* is the total sum of squares: sum [(true position–mean (true position))^2^]. This metric thus ranges between zero and one for optimal performance.

To verify the suitability of different electrode configurations in more detail, the deviation between the predicted values of the test set and the true virtual fish size, position, and orientation was calculated. In the following, we focus on the position error as we only used this in the behavioral experiments. Matplotlib (Version 3.2.1) was used for visualizing the results (Hunter, 2007).

### Using Supervised Learning to Predict Fish Position of Real Fish

**Figure 2:**
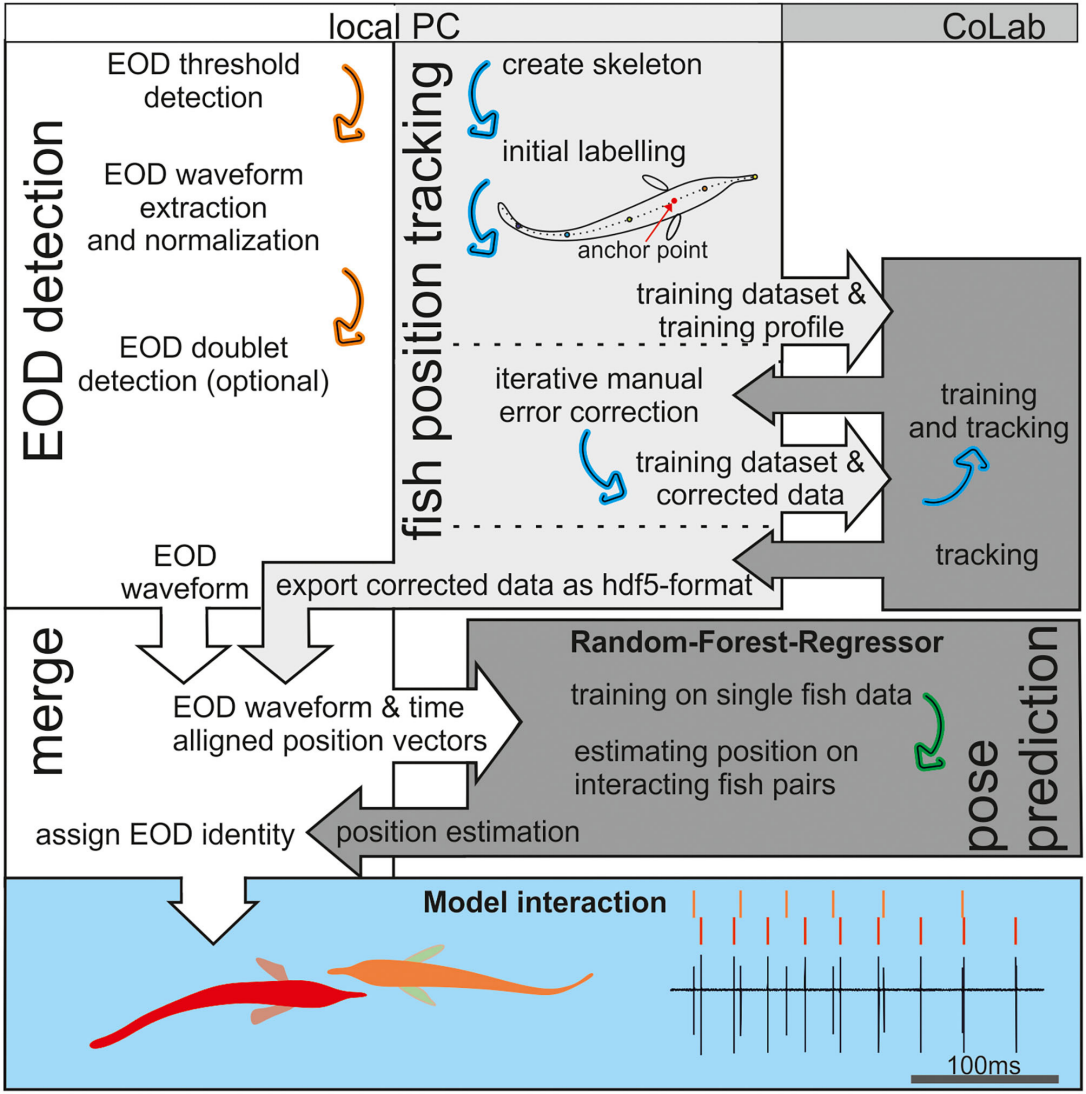
An Illustration of the workflow. Electric organ discharge (EOD) detection was done in Matlab (white background), whereas fish tracking and pose estimation were based on Python (gray). The tracking in the open-source package social leap estimates animal poses (SLEAP) is based on deep neuronal networks that are computational demanding. Therefore, we relayed the training and final tracking of the data onto the CoLab platform (medium gray). The RFR (dark gray) was trained using the EOD waveform vectors as an input and the tracked (single fish) position data as the target. After training, the EOD waveform vectors were used to estimate fish position. This estimate from the RFR then was compared to the tracked fish position to assign the EOD identity in Matlab (see also Figure 7). The later behavioral analysis of agonistic behavior relied on the data set generate in this way to analyze the electrosensory information provided by the active and passive electrosensory system during the observed behavior (blue background).

We used supervised learning methods to predict fish position based on the recorded EOD data with the aim to assign EODs to individual fish. The workflow (Figure 2) consisted of several interacting steps as described in detail in the following.

The RFR model was used again for the behavioral data; in addition, we also tested different MLP models. There were a few changes with respect to the setup and the data estimated. Fish were allowed to move freely within the experimental tank, including tank boundaries. Furthermore, we did not estimate the virtual fish position and orientation, but rather the position of the six nodes of the skeleton of an animal (Schnauzenorgan, head1, head2, mid1, mid2, and tail) as previously defined in SLEAP (see section Fish Position Tracking). We used the default parameter settings of the RFR model from the Scikit-learn package, also the “n_estimators” was set to 100.

We used an MLP model from the Scikit-learn package (Version 0.22.2) with default settings (except for the two parameters) to construct neuronal networks of different size. The size of the hidden layer was 50, 100, or 200, respectively. The number of iterations was 10,000 to ensure that all models converged (Pedregosa et al., 2011).

**Figure 3:**
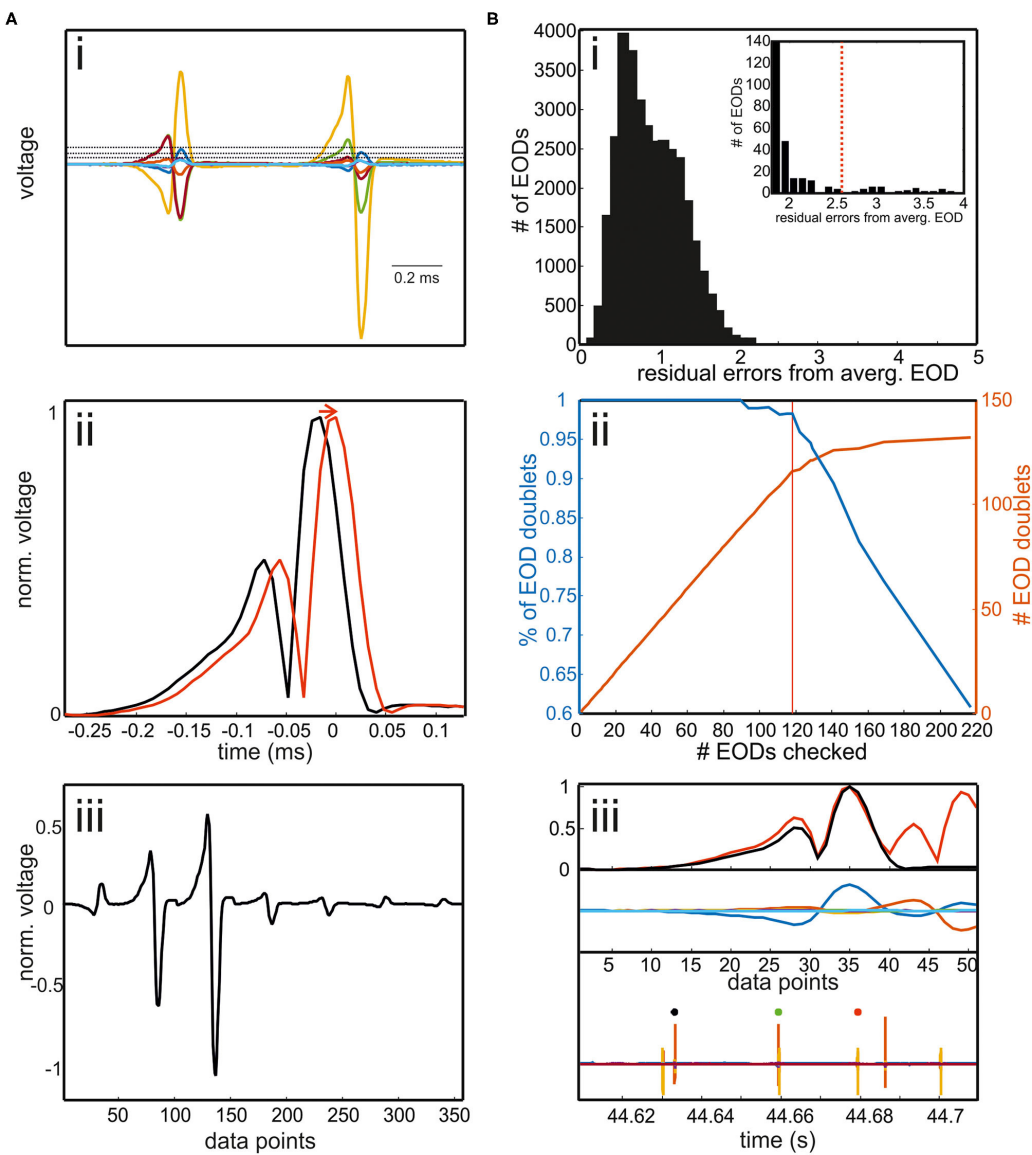
**(A)** Extracting EOD input vector for the RFR model. **(i)** EODs were extracted by a threshold operation. The threshold was defined differently for each channel. It could be set way above noise level as EODs had to be detected only in one of seven electrode recordings. If two EODs were separated in time by less than 400 μs, the later EOD was deleted. Interfering EODs of two fish were detected in an additional step. **(ii)** To align EODs, we took the absolute values and averaged across all electrodes. EODs were cut 272 μs before and 128 μs after the EOD peak. **(iii)** The concatenated EODs were stored as a vector. **(B)** Detection of interfering EODs. **(i)** Histogram of the residual errors of single EOD waveforms from an average EOD waveform. EODs were normalized to the absolute maximum peak beforehand. Inset: enlargement of the histogram including the threshold above which EOD waveforms were checked. **(ii)** Percentage of EOD doublets of the total number of EODs checked (blue) and the absolute number of EOD doublets found (red) plotted against the number of EODs checked. With the threshold shown in red in the inset in [**B(i)**], 119 EODs needed to be inspected. Of these 98% are doublet EODs, which accounts for almost 90% of all doublets in the data set. Thus, the manual re-analysis can be made very efficient by concentrating on this “suspicious” fraction of the total data. **(iii)** Example of two overlapping EODs. EOD doublet can be easily identified by visual inspection. Upper panel: normalized average EOD waveform (black) and single EOD waveform (red); middle panel: EOD recordings aligned to the maximum peaks. Lower panel: EOD recordings including the EOD detection surrounding the current “doublet” EOD (green).

**Figure 4:**
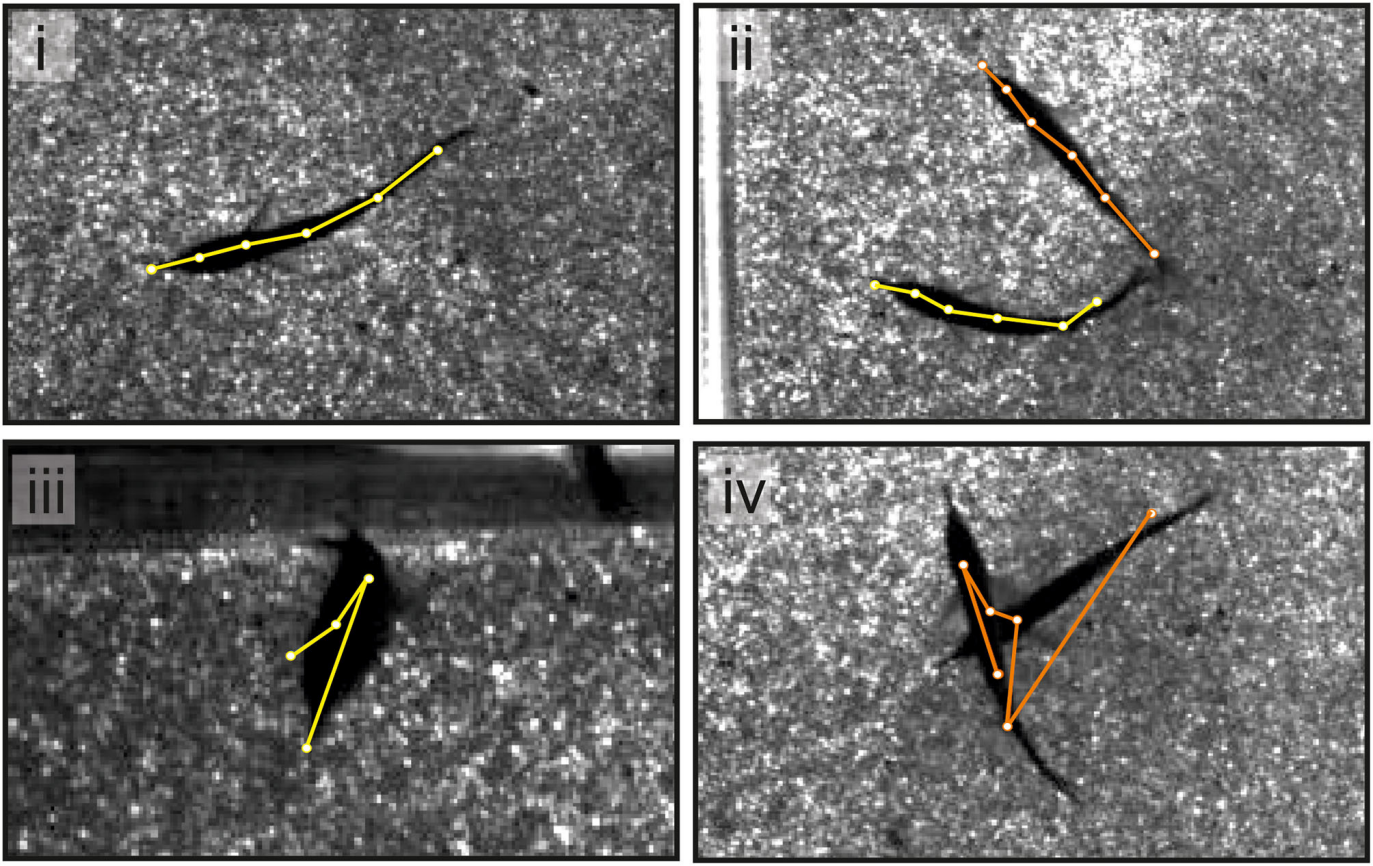
An example of single video frames shows the superimposed skeletons tracked using SLEAP. In the majority of cases (see **i,ii**), the tracking using SLEAP was sufficiently precise. When tracking a single fish, the error rate was low and errors occur when fish took unusual postures (see **iii**). For dyadic interactions, errors were more frequent and dependent on the distance between animals (see **iv**).

To train the supervised learning models, 40 videos from the data set I with single fish swimming in the area were used (58.385 EODs and corresponding fish positions). The EOD waveform vectors [see section EOD Detection; Figure 3A(iii)] were used as the input, and the positions of the six skeleton nodes of the fish midline (Figure 4) were the target to be learned. The performance between models and model configurations was assessed using 25% of the data previously not included in the model training. From the four options, the RFR model was the best in localizing the position of the emitting fish as verified using the head position of an animal now (Figure 1C). This model thus was used on data set I with both fish swimming together (15.763 EODs and positions). While the network estimated the position of six nodes for each individual, we are primarily interested in the identity of the EOD-emitting fish. Therefore, we calculated the root mean square (RMS) error (summed over all six nodes) between the model estimated fish positions of the true fish positions (**Figure 7A**). The fish with the smallest RMS error was labeled as the EOD-emitting fish.

To compare this automated identification to the human-observer identification, we first manually assigned all 15.763 EODs based on visual inspection to the individual fish. To estimate the precision of this human-observer approach, the process was conducted a second time after 3 months, and a disagreement in about 1% of the cases was found. Because the error rate using the network was substantially larger than 1%, we manually corrected those EODs presumably wrongly assigned by the model.

### EOD Detection

For EOD detection, a custom written Matlab program was used (Figures 2, 3). To obtain the EOD vector later used for EOD assignment in the supervised learning models, the following steps were taken (Figure 3A):

- *Electric organ discharge threshold detection*: EODs were detected using a separate threshold for each of the seven channels. The thresholds were set such that they were clearly above the noise level and low enough that each EOD was detected at least from one channel.
- *Waveform extraction*: to temporally align the EODs, we took the average of the absolute values on all channels [Figure 3A(ii)]. Then, the original EOD channels were aligned to the maximum peak of the mean absolute trace. We cut individual EODs using a temporal window of 272 μs before and 128 μs after the peak.
- *Waveform vector normalization*: a row vector was created containing the data of an EOD of all seven electrodes. This row vector was then normalized by the absolute maximum peak resulting in a voltage range of ±1 V [Figure 3A(iii)]. This step was taken to make the EOD assignment more robust against variable fish sizes.

Temporally overlapping EODs of two fish were detected in the following way (Figure 3B):

iv) *EOD doublet detection*: each normalized EOD waveform was automatically compared to the average normalized EOD [Figure 3B(iii) top]. To obtain the normalized average EOD waveform, the absolute values of the EOD recordings were averaged across all detections and channels and then normalized by dividing the average by the peak amplitude. Correspondingly, the normalized EOD was obtained by averaging the absolute values of the seven EOD channels and then normalized by the maximum.

The histogram of the difference between the normalized single EOD waveforms and the normalized average EOD waveform was long-tailed in each data set. The majority of EOD doublets was found in this tail of the histogram [Figure 3B(i,ii)], allowing to focus the manual correction to a fraction of the actual data: if the absolute deviation exceeded a threshold, EODs were visually inspected by the user to determine if doublets occurred [Figure 3B(iii)]. We stored the time point of the doublet and reassigned the EOD to both fish after the automatic assignment of the EODs with the RFR model.

### Fish Position Tracking

For video-based tracking of the fish positions, we used SLEAP (Pereira et al., 2019, 2020; Figures 2, 4). SLEAP is a framework for multi-animal body part position estimation *via* deep learning. It is written in Python and comes with a labeling and training GUI that supports active learning. We used the multi-animal top-down approach because an initial comparison showed better results than the bottom-up approach also available in this framework. The top-down approach complements two different models: the centroid model first predicts the location of each animal in a given frame. Then, the instance centered confidence map model is used to predict the locations of all the nodes (“posture”) for each animal separately.

The use of SLEAP is well documented elsewhere (https://sleap.ai) (Pereira et al., 2019, 2020). However, in short, our workflow consisted of the following steps (Figure 2; light and medium gray panels).

- *Creating the skeleton*: the skeleton consisted of six user defined nodes (e.g., Schnauzenorgan, head positions 1 and 2, mid positions 1 and 2, and tail) and the corresponding edges (e.g., connection between Schnauzenorgan and head position 1).
- *Initial labeling*: about 50 randomly chosen frames out of 10 videos (*data set I*, two interacting fish) were used for the initial labeling. To account for a variation in the setup, we chose the videos where the background differed, i.e., the glass beads covering the floor were manually shuffled, resulting in a heterogeneous distribution of the background intensity.
- *Creating a custom training profile*: the SLEAP-label GUI was used to create a custom training profile for the multi-animal top-down model. The profile must be adjusted to match the animal under investigation. Specifically, we adjusted the anchor part and the input scaling. The anchor part is an important parameter as it is used to estimate where each animal is located. We chose head position 2 as the *anchor part* as it was in a relatively central position and can be precisely located as the midpoint between the pectoral fins. The input scaling of the centroid model was set to 0.5, which enabled matching of the receptive field size to the actual fish size. In addition, the input scaling of the centered instance model was set to 1.25, to obtain a smaller receptive field than for the centroid model.
- *Training*: the customized training profile and the training data set were transferred to Google Colab. Training was then run in Google Colab using a Python script based on the example notebooks for top-down models (function: SLEAP-train; profiles: centroid.json and centered_instance.json). We used “flow” as the tracking method. Here, SLEAP takes instances from the prior frames. Then, points in the instance are shifted based on the use of optical flow (Xiao et al., 2018). These shifted points are used as the candidate instances.
- *Retraining*: after training, the network was used to predict the skeleton positions of the fish for 5 of the previous 10 videos. These predictions were transferred back to our local PC. We manually picked 10 frames per video where the quality of the labeling was low. Incorrect labels were corrected, and a new training data set was created, including the previously randomly chosen 50 data frames and now selected and corrected 50 data frames. In this way, the network got randomly chosen data as an input as well as the data that were specifically hard to allocate correctly. This data set was again transferred to Google Colab, and the model was retrained. Afterward, following the abovementioned procedure, the remaining five videos were used to add more labels to the training data set. In a total of 150 frames that were used to train the network, 50 of those were randomly chosen, and 100 were chosen based on the detection performance of the neuronal networks from the different training iterations.
- *Analyzing videos*: following steps i–v, we analyzed all videos (single- and multi-animal videos) using Colab. The obtained videos were then further processed using the SLEAP-label GUI to correct the remaining errors on local PCs (Figure 4). The results were then saved in hdf5-format for further processing in Matlab.

### Dyadic Interactions

To study dyadic interactions, fish were placed on opposite sides of the experimental setup. A gate prevented them from entering the inner part of the arena, and the gates were operated remotely and opened simultaneously after an acclimatization time of 10 min. Behavior was videotaped at 30 frames/second for the first min following gate opening, and the first contact behavior was analyzed based on these videos. First contact was defined as the first time fish touched each other. EIs were calculated from the simultaneously recorded EODs.

Within the first 10 min after the first contact, a 5-min video was recorded at 10 frames/second. This video was used to evaluate the fight resolution. Fight resolution was determined by counting the number of attacks and observing the chasing behavior from these videos. An attack was defined as a contact between both animals in which at least one individual changed body posture as a result of the contact. Chasing was defined as one fish being followed by the other fish using approximately the same trajectory.

### Modeling the EIs

Passive and active electroreception is mediated through different electroreceptors that both occur and are distributed over the skin of an animal. The electric pattern, i.e., the distribution of local field intensities, provides the relevant input to both modalities. This spatial distribution is referred to as the EI (Caputi and Budelli, 2006). To distinguish between active and passive sources, we will refer to the sensory images generated by the presence of external electric fields as passive EI and to those images generated by the distortions through elements in the electrosensory scenery as active EI.

Electric images were computed with the software originally developed by Rother (2003). This approach was verified and utilized in previous studies (Rother et al., 2003; Migliaro et al., 2005; Sanguinetti-Scheck et al., 2011; Hofmann et al., 2013, 2017; Pedraja et al., 2014). The model estimates the transcutaneous current density for each of the points on the surface of the animal and is based on the following assumptions:

(1) All media are ohmic conductors. This means that the vector representing the current density at the point *x* (*J*(*x*)) is proportional to the vector electric field at the same point *E*(*x*). Then,

(2) $$\begin{array}{l} \text{J}\left(\text{x}\right)=\sigma \left(\text{x}\right)\text{E}\left(\text{x}\right),\sigma \left(\text{x}\right)>0. \end{array}$$

(2) The proportionality constant σ(x) is the volumetric conductivity at the point *x*.
The model neglects capacitive effects, that is, we assume that there is no accumulation of charge p(x) at any point in space.

(3) $$\begin{array}{l} \delta p\left(\text{x}\right)/\delta \left(\text{t}\right)=0. \end{array}$$

(3) Given that the dielectric relaxation of the media in general is shorter than the minimum significant period of the EOD Fourier components, the model is an electrostatic approximation (Bacher, 1983).
(4) The space is divided into volumes of homogeneous conductivity. The fish and the different objects are defined as the zones of different conductivity immersed in an infinite water medium. Each object is covered by a thin resistive layer (the skin in the case of the fish), which can be homogeneous or heterogeneous (magnitudes specified as desired).
The model is based on the charge density equation which, under the above assumptions, implies that the charge generated by the sources *f* (*x*) is equal to the charge diffusion:

(4) $$\begin{array}{l} \delta p\left(\text{x}\right)/\delta \left(\text{t}\right)=\text{f}\left(\text{x}\right)-\nabla \cdot \text{J}\left(\text{x}\right) \end{array}$$

Using Equation (3) and then Equation (2)

(5) $$\begin{array}{l} \nabla \cdot \text{J}\left(\text{x}\right)=\text{f}\left(\text{x}\right)\Rightarrow \sigma \nabla \cdot \text{E}\left(\text{x}\right)=\text{f}\left(\text{x}\right) \end{array}$$

The electric field E(x) can be expressed as *E*(*x*) = –∇φ, therefore,

(6) $$\begin{array}{l} \sigma \nabla^{2}\varphi \left(\text{x}\right)=-\text{f}\left(\text{x}\right), \end{array}$$

where φ(*x*) is the local potential at point *x*.

Equation (6) is a partial differential equation known as the Poisson equation and can be solved for every point in space, in our case the fish boundaries by using the boundary element method (BEM) as proposed by Assad (1997). For a formal explanation of the BEM (please see Assad, 1997; Hunter and Pullan, 2001; Rother, 2003; Brebbia et al., 2012). Briefly, this method determines the boundary conditions by solving a linear system of *M* · *N* equations for *M* poles and *N* nodes, with the unknown variables being the transepithelial current density and potential at each node (Pedraja et al., 2014). The transepithelial current density and potential are calculated for each node and linearly interpolated for the triangles formed by the nodes. The choice of nodes allows for an approximation of the shape of objects and fish, and by scaling the number of nodes the spatial resolution of the EIs can be chosen to match the computational power available. We based our model on a set of 49 ellipses composed of 17 nodes each (835 nodes forming 1,666 triangles) (Rother, 2003). The size of the fish can be scaled by changing the two diameters of each ellipse and changing the distance between ellipses.

**Figure 5:**
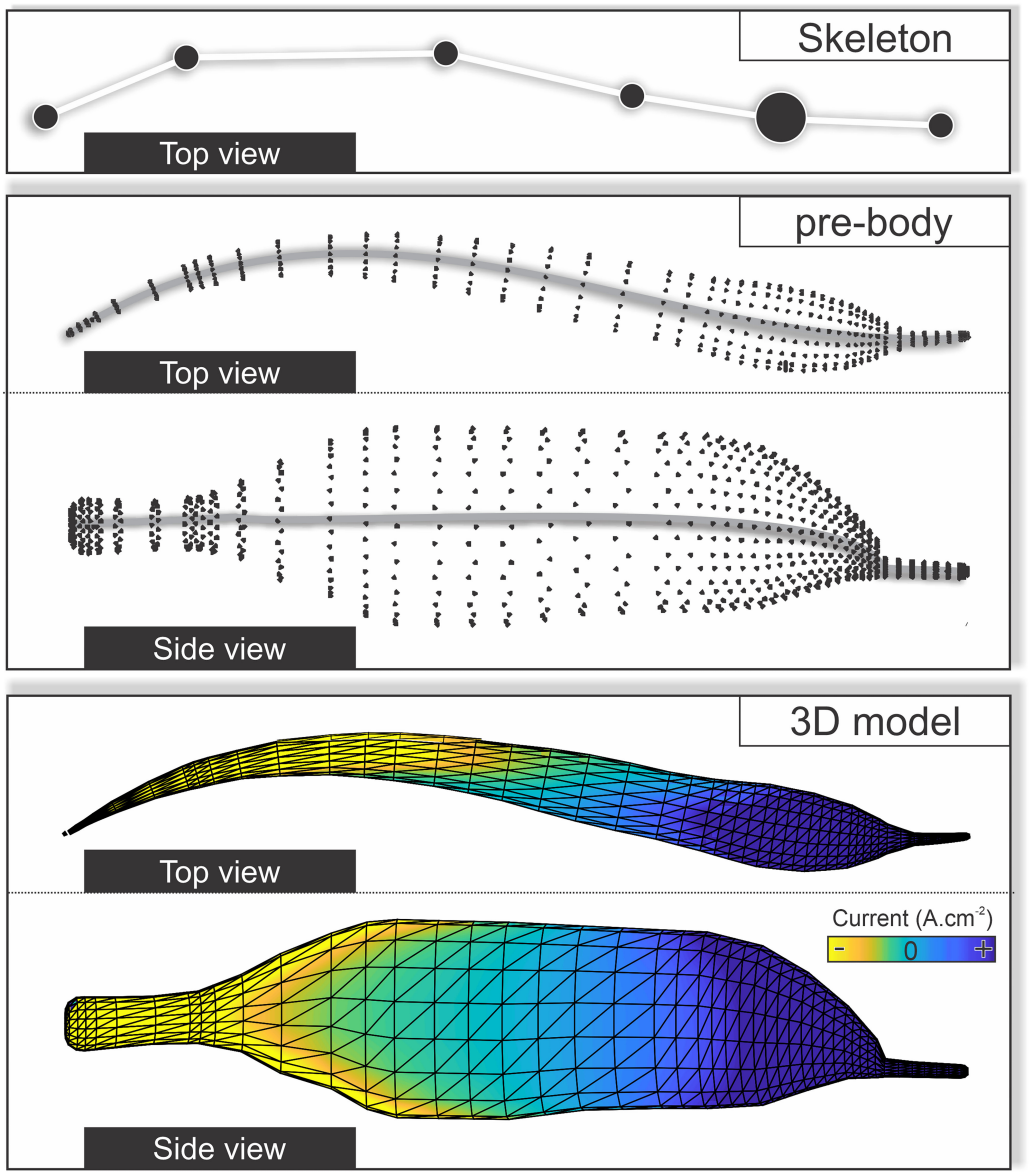
The transition from the fish skeleton estimated by SLEAP to a three-dimensional (3D) model was used to model the electric images (EIs). The skeleton obtained from the tracking procedure is shown in the upper panel and is fitted by a third-order polynomial. A set of pre-defined ellipses that form the body of the 3D fish are then distributed along the polynomial (middle). Note that the ellipses can be scaled in size and distance to match the true size of the fish. The points of the ellipses represent the nodes that are connected to form a total of 1,666 triangles for which we then calculated the EIs using a well-established boundary element method (BEM) (bottom).

To adjust the model to the actual fish posture, a third-order polynomial was fitted to the six skeleton points of the actual fish (see above). The fish-body ellipses were realigned according to the first derivative of the fitted polynomial to match the rotation of the ellipses to the curvature of the posture of the fish. From this, nodes and surfaces are produced to result in the final three-dimensional (3D) reconstruction for which the electric current and the transcutaneous voltage were calculated (Figure 5, Hofmann et al., 2014; Pedraja et al., 2020).

For our analysis, we separated passive and active EI information. This basically means that for each EOD we calculated the resulting EI perceived by the EOD-emitting fish (active EI) and the EI provided to the non-emitting receiving fish (passive EI). This was done for all EODs up to the first physical encounter of the fish. Active EIs were calculated as the difference between the electrosensory stimulus (scene with both fish) and the basal field (scene with just the discharging fish) at the discharging fish sensory surface. Passive EIs were obtained by using the electrosensory stimulus at the sensory surface of the nondischarging fish (Pedraja et al., 2016). Based on the EIs, we estimated which fish could have obtained sufficient information to detect the other fish first. For this, we consider the moment where the EI amplitude of one fish exceeded that received by the other fish by a factor of two. This was independently determined for both passive and active EI. This analysis was limited to inter-fish distances where EI amplitudes exceeded currents above 0.1 μA as this is the internal noise range of the model.

## Results

In the following, we will first detail the steps we took in optimizing the EOD recording setup using supervised learning methods to then demonstrate how two different models performed in automatically localizing an EOD-emitting fish in the setup.

To further demonstrate the robustness of the approach we found to be best suited, we then will report data where the RFR model approach is being used on dyadic interactions of fish to investigate if and how the RHP may be perceived and used in conflict resolution between dyads of *G. petersii*.

### Using Supervised Learning to Predict Fish Position and Orientation of Simulated Data

**Figure 6:**
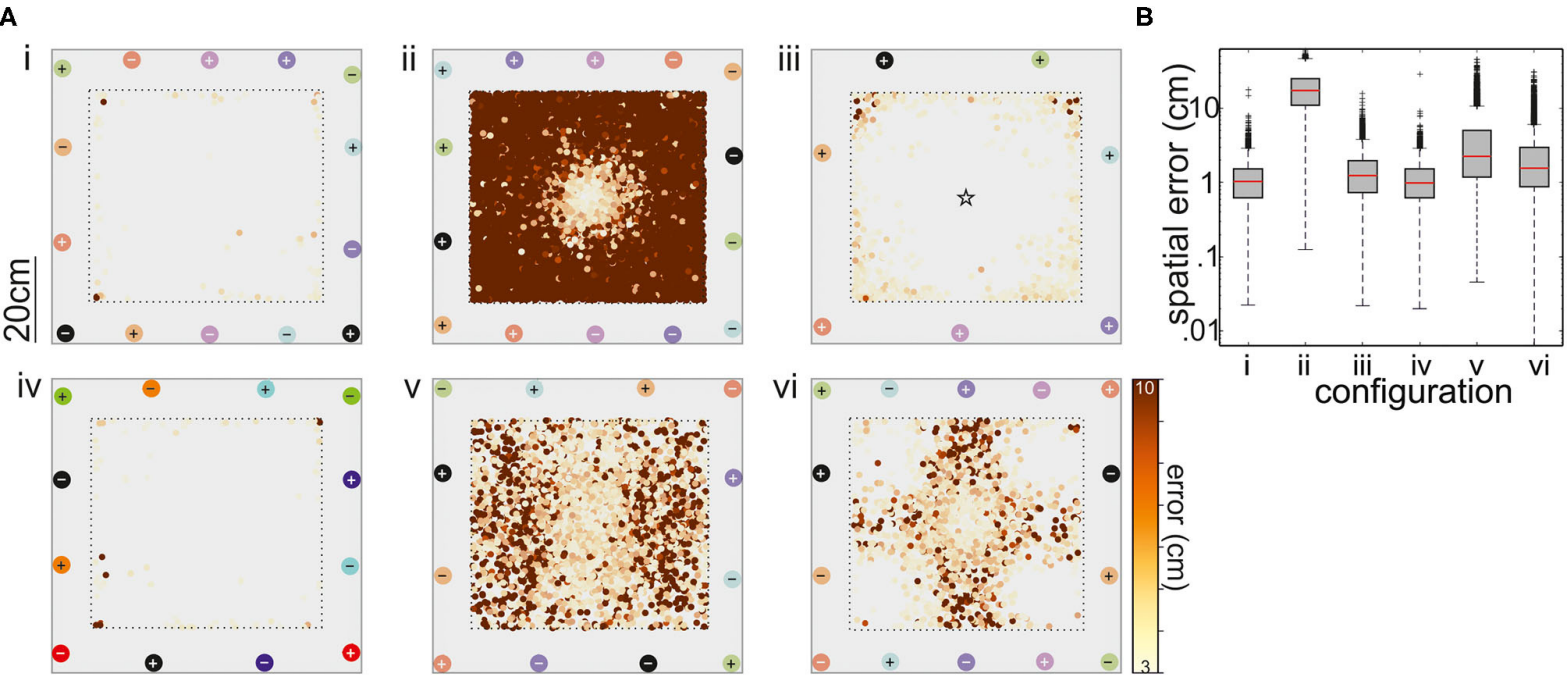
**(A)** Performance of the tracking with respect to six different electrode configurations. Electrode pairs are marked with different colors, where + corresponds to the positive and – to the negative pole. With the exception of the arrangement shown in **(iii)**, all recordings were differential (refer to matching colors of electrodes, depicted as circles). In the setup shown in panel (iii), the ground electrode was situated in the middle of the tank. The top view of the arena shows where tracking errors larger than 3 cm occurred. The magnitude of these errors is color coded (see bar). **(B)** Box-and-whisker plots of the spatial error for the six electrode configurations. The configurations 1, 3, and 4 had the best performance and configuration 1 was further used in the behavioral experiments on dyadic interactions (see text for details).

The positioning of the electrodes in the experimental arena is important to obtain suitable coverage of the EODs and sufficient resolution to distinguish EODs between individual fish. By combining a simulated electric field to mimic a swimming electric fish with a supervised learning-based algorithm that predicted the position of this fish mimic, we compared six different electrode arrangements, including both differential and unipolar recordings (Figure 6). For this, electrode arrangement along the tank walls was altered. We did not test electrodes on the floor as this would have interfered with the video recordings, but the methodology of optimizing the electrode arrangement described here is applicable to arbitrary experimental layouts and electrode configurations.

The degree to which the supervised learning model was able to correctly locate the fish mimic was used to obtain the best of the six arrangements tested. Configurations 1–6 are shown on Figure 6A(i–vi), respectively. With one exception, the model performance was good, as indicated by *R*^2^ scores >0.9. The exception is the arrangement two. Here, the axis of neighboring (differential) recording pairs was continuously rotated from one pair to the next, leading to a comparatively good resolution in the middle of the tank, while positioning was poor toward the tank walls. A similar arrangement (configuration 3) with the reference in the center of the tank improved the performance, particularly avoiding errors between fish positions at opposite electrodes, but still mislocating the mimicked fish toward the walls. Similar problems occurred for the configurations 5 and 6. Configuration 6 used the five electrode pairs that form a perpendicular net, whereas two electrodes pair have a different angle by crossing from corner to corner. This resulted in satisfactory spatial resolution at the corners and the center of the tank but in larger errors in the remaining arena.

In summary, configurations 1, 3, and 4 are determined as suitable electrode configurations, in the majority of mimic positions and orientations, the mislocation of the mimic EOD was below 2 cm (Figure 6B). Given that the fish are at least 10 cm in length, an error of this magnitude was considered acceptable. Fish tend to spend much time at the tank walls and corners (personal observation; Teyke, 1989), configuration 3, where errors were particularly high for the corners of the tank, was also discarded. Configurations 1 and 4 were comparable, we chose configuration 1 for all the following experiments. With seven electrodes, we assumed (though we did not test this) that we might have a slightly better performance close to the borders of the setup when working on real data as there is a higher spatial sampling of EOD data.

### EOD Detection

The analysis of EOD data involved detecting the time point of the event and extracting the EOD waveform in a standardized manner (Figure 3A). Both are needed as an input for our RFR model to estimate fish position from the field geometry. As the initial training of the network was based on the data with a single fish in the tank, it could not correctly assign EODs of two fish that occurred simultaneously within the time window of 400 μs used during the training. EOD doublets were reliably found by looking at the deviation of the EOD waveforms from the average EOD waveform (Figure 3B). False EOD doublet detections typically occurred when the electric potentials were low on all electrodes. This process could also be fully automated in the future. Note, however, that the used algorithm is limited to dyadic interactions.

### Tracking With SLEAP

The performance of the tracking model was different for single- (*n* = 72,000 frames) vs. multi-animal videos (*n* = 18,000 frames) (Figure 4). Tracking of single animal was efficient. The fish was detected in all frames but one and the body posture needed to be corrected in <2% of the frames (Figure 4, bottom left). Corrections to the tracking were thus limited and mainly restricted to situations where the fish made unusual movements, e.g., tried jumping. With two fish in the arena, one of either fish was not detected in less than 1% of the frames. This was typically the case when fish were very close to each other or partially overlapped (Figure 4, bottom right). The posture, i.e., the six nodes of the midline of the animal needed adjustment in about 5% of the frames.

### Using Supervised Learning to Predict Fish Position of Real Fish

**Figure 7:**
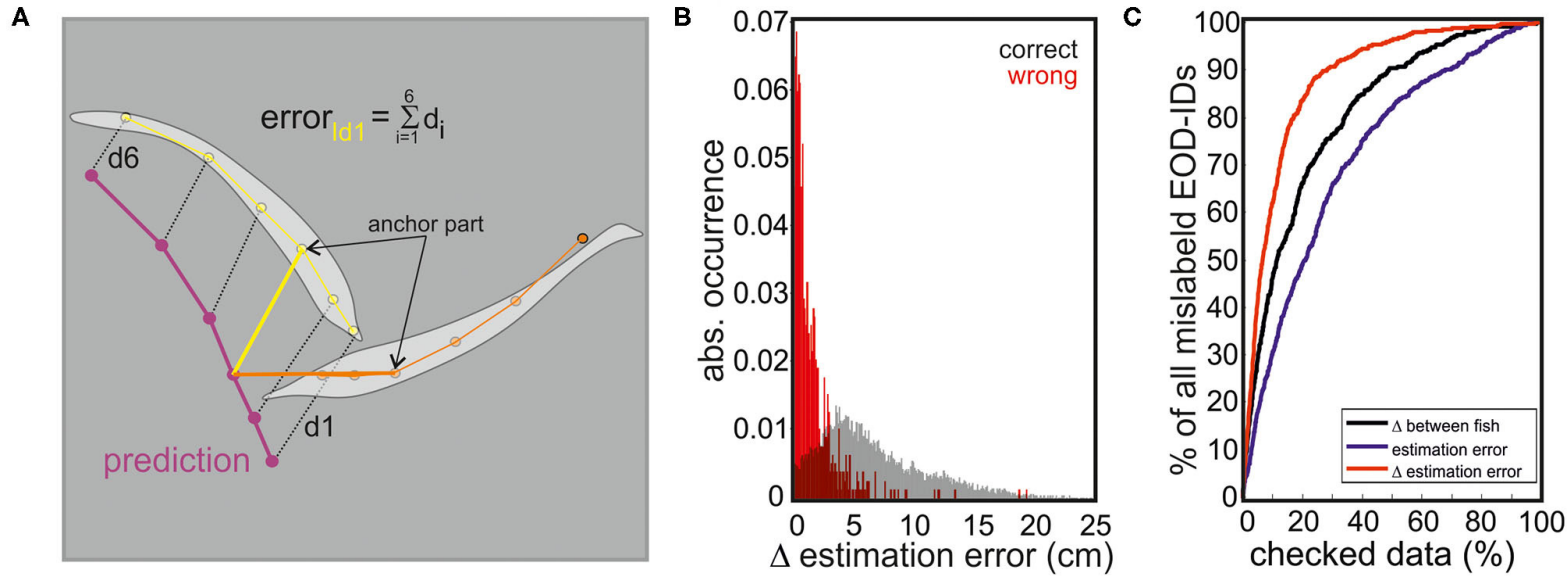
**(A)** Estimation error calculation. The distance between each skeleton node of the predicted fish (based on the RFR) and the tracked fish (based on SLEAP) was measured. The predicted fish location that had the smallest sum of distances from either of the two tracked fish (estimation error) was considered as the EOD producing fish. **(B)** Histogram of correct (black) and incorrect (red) identity assignments as a function of the difference in estimation error of both fish (abs(errorID_1_-errorID_2_, red)). **(C)** We tested different parameters to assess the likelihood that the model erroneously assigned EODs to individuals. The first parameter used was the distance between individual fish (black), the second the estimation error (blue), and the third the difference between the estimation errors of both fish (red). The percentage of mislabeled EOD-IDs found during manual re-inspection of the data is plotted against the percentage of data that had to be checked for different measurements. Reading example: checking 5% of the data using a threshold criteria based on the difference between both fish resulted in a capture of 30% of all errors, or in other words the 5% of the data that had the smallest difference between both fish contained 30% of all mislabeled EODs. When checking 5% of the data using a threshold criteria relying on the estimation error alone allowed an identification of only 18% of all errors. However, when checking 5% of the data using the threshold criteria based on the difference in estimation error, 40% of all errors could be found. Hence, using the threshold criteria based on the difference in the estimation error is the most efficient way to find wrongly assigned EODs.

We tested an RFR model and different MLP models of various numbers of hidden layers to predict the fish position from the EOD data. Using the single fish recordings from data set I, we could establish how well the models fared in predicting the fish position. For simplicity, we now only considered the error in the anchor point (second head node, see Figure 7). The error that is the distance between the tracked anchor point and the EOD predicted anchor point was the smallest (median: 1.48 cm) for the RFR model but the MLP model with 200 hidden layers had an almost comparable performance (median: 2.18 cm). Given its performance, we chose to continue with the RFR model only.

Indeed, the RFR model was further found to be valuable in identifying the EOD-emitting fish in dyadic interactions (data set I). Here, the performance that is correctly labeling EOD-IDs (and the localization of the emitting individual) was around 95%. Errors mainly occurred for short distances between individuals (Figure 7C) and are also dependent on fish positions.

With this, it was possible to further improve the performance focusing on the data most likely to cause problems. Therefore, we calculated the difference of the estimation error for both fish (see Figure 7A). By focusing on the 5% of the data where the difference in the estimation error was the smallest, 40% of all wrong assignments were found, enabling us to manually improve the total error to <3%. The inclusion of 10% of the data with the lowest difference in the estimation error resulted in an assignment error of 2%.

Given the performance of the model and the enormous reduction of observer-based annotation time, we applied the workflow established now to dyadic interactions of new fish pairs that differed in size from the fish used in training the network (data set II). In addition, the interactions studied now were limited to the initial approach phase, where the inter-fish distances and relative orientations presumably will be different from the training conditions. Despite these differences, the performance was found to be robust: less than 6% (*n* = 348) of the EODs (*n* = 5,848) were wrongly assigned (in 6% of all EODs a human observer chose a different EOD-emitting fish).

### Dyadic Interactions

**Figure 8:**
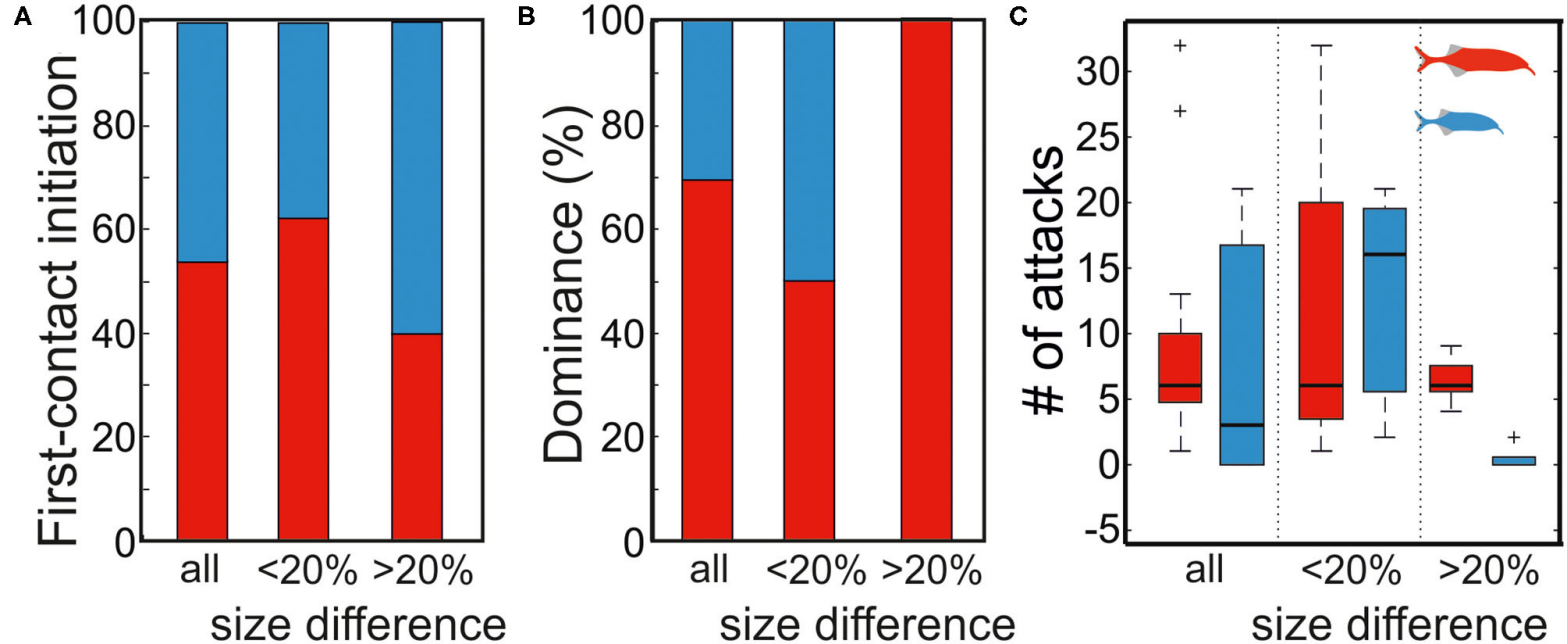
Effect of fish size on first contact initiation and dominance status. **(A)** The first contact was initiate independent of fish size (binomial test, *p* = 0.5, 0.5, and 0.36 for all, >20%, and <20% size differences, respectively). **(B)** The dominance status dependent on fish size for size differences >20% (binomial test, *p* = 0.031). No difference in dominance was found for small size differences (binomial test, *p* = 0.636). **(C)** Aggression level also was dependent on size difference. Big fish attacked significantly more during dyadic interactions of large size differences while more aggressive behaviors (number of attacks) were found in an interaction among fish of small size differences. Red and blue symbols represent the large and small fish, respectively.

About 13 pairings of fish that had not physically encountered each other before were analyzed. In five pairings, the difference in body size was 23–28%, whereas in the remaining eight pairings the size difference was between 2 and 17%. We found no dependency between the fish size and the animal that initiated the first encounter (binomial test; for all pairings 7 (larger) fish in 13 pairings initiated an attack, *p* = 0.5; for length difference <20% 5 (larger) fish in 8 fish pairings initiated an attack, *p* = 0.36, and for length difference >20% 2 (larger) fish in 5 pairings initiated an attack, *p* >= 0.5; Figure 8A).

Based on the number of attacks initiated by an individual, we determined the dominance status within each pairing (Figure 8B). In pairings with a large size difference (>20%), the larger fish was always the dominant individual, whereas the outcome of the encounter was not predictable in the smaller size difference pairings (binomial test; 5/5, *p* = 0.031 for length difference >20% and 4/8, *p* = 0.636 for length difference <20%, Figure 8B). Considering all pairings, no significant relation between size difference and dominance was found (binomial test; 9/13, *p* = 0.133). We also measured the time individuals spent in chasing each other. Aggression, as now measured by the number of attacks a fish does, overall was higher in dyadic interaction of low size differences (Figure 8C), whereas in interactions of larger size differences there were fewer attacks that were predominantly executed by the larger fish (Figure 8C).

### Initial Approach Phase

**Figure 9:**
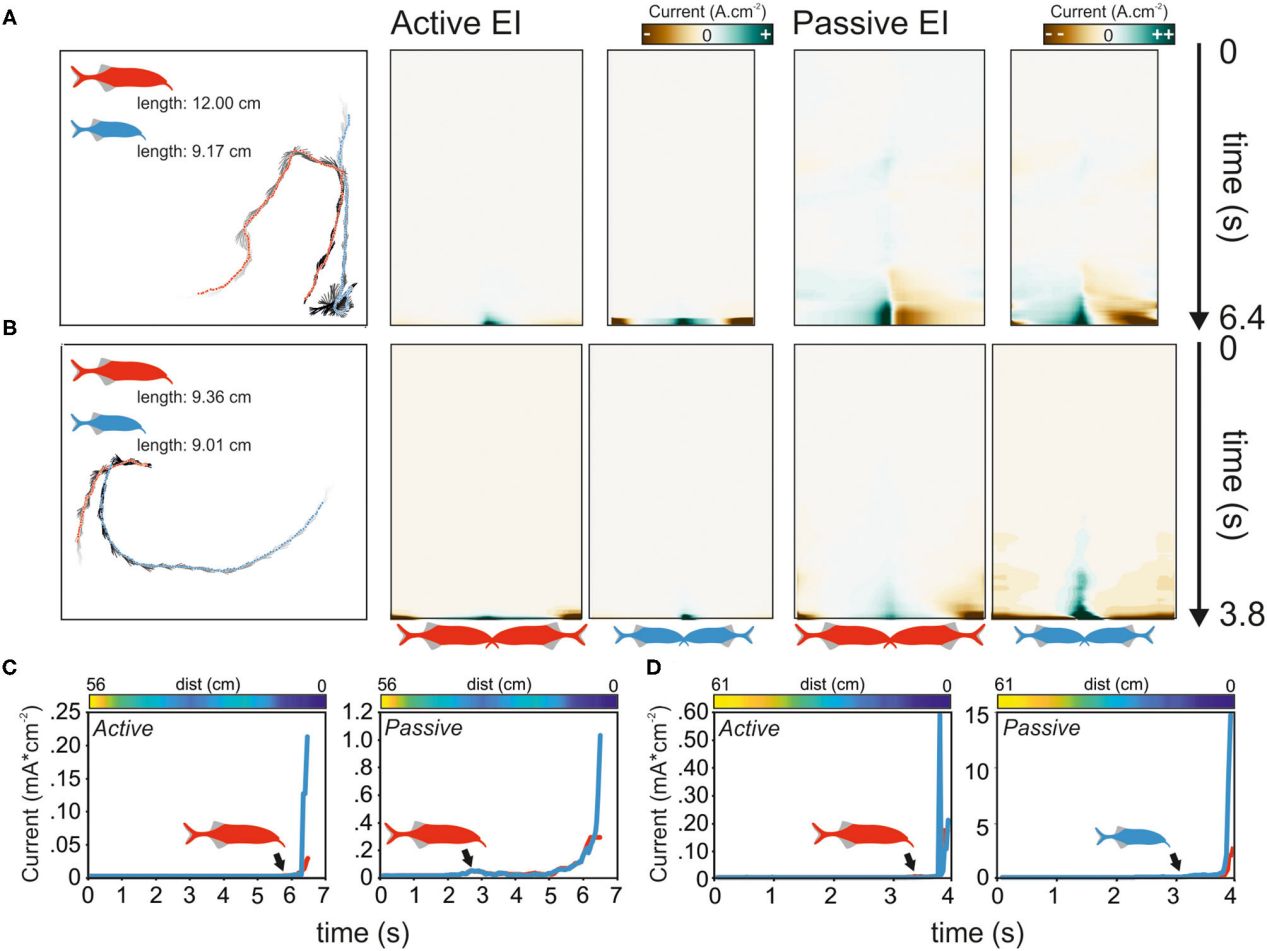
Analysis of electrosensory input calculated for the initial approach phase during the agonistic encounter of dyads of *Gnathonemus petersii*. **(A,B)** Left: approach trajectories of two fish of large **(A)** and small size differences **(B)**. Points indicate the fish positions and gray lines indicate their orientations. Time from start to the first physical encounter is depicted by the gray gradient of the orientation labels (light gray = start). Middle: color-coded RMS of the active EIs of the sequences are shown on the left. Here, zero on the y-axis indicates the start of the experiment (see black arrow), and the images end with the first contact. Right: same as data on the left but for passive EIs. **(C,D)** The maxima of active (left) and passive (right) EI of both fish are plotted against time. The distance between individuals is shown in the color-coded bar above the plot. Blue colors indicate close distances, whereas yellow colors represent larger distances. Note that in C the distance does not constantly reduce over time. The black arrows indicate the time point at which the EI of both fish start to diverge. The fish perceiving the strongest EI is shown in a color-coded fashion. The data in C corresponds to the approach depicted in **(A)**, whereas the data in **(D)** refer to the approach depicted in **(B)**. Red and blue symbols represent the large and small fish, respectively.

Regardless of the size difference, the approaching fish always initiated the first contact. In 9 of the 13 interactions, these contacts were directed toward the tail of the opponent, in the remaining 4 cases, the head was targeted. To understand if the electric sense contributes to the behavior and whether active or passive electrolocation is used, we next modeled the electrosensory input for each first-approach trajectory. Figure 9 shows the results of the EI reconstruction for two exemplary dyadic interactions. While Figures 9A,C depict the data for a dyad with a large size difference, Figures 9B,D show the result for a small size difference interaction. Although the trajectories leading to a contact varied between fish, we frequently observed that the passive and active EIs peaked on the head and tail regions, almost irrespective of the relative orientation between contenders (e.g., Figures 9A,B right).

Independent of the size differences, the passive EI could best explain which fish approached first. In 10 of the 13 cases, the fish initiating the attack is predicted to having perceived the contenders passive EI before the contender would have detected the approaching fish (binominal test; *p* = 0.046). In contrast, the active EI was only more intense in the approaching fish in 6 of 13 cases (binomial test; *p* >= 0.71). With respect to size differences, the passive EIs were the largest for the attack leading fish in all cases for the >20% group (binominal test; 5/5, *p* = 0.031). Meanwhile, this was not the case in the smaller size difference pairings (binomial test; 5/8, *p* = 0.363). This indicates that the information obtained from the passive EI guides the approach leading to the first contact.

## Discussion

We now introduced the use of supervised learning models to aid in the study of (pulsatile) weakly electric fish and applied the methodology to investigate the role of electrosensory information in the dyadic interactions of *G. petersii*. Our initial objective was to introduce a workflow to overcome the bottleneck that the time-consuming human-observer-based allocation of EODs to individual fish poses to the study of electrocommunication, especially in pulse-type species. For this, we first optimized the recording configuration by combining a simple electric field model that mimicked the electric field of the fish with an RFR model from the open Scikit-learn package. By combining the open-source tracking tool SLEAP with an RFR model, we then showed that the EOD data recorded using the recording configuration we had determined as the best for our setup can be used to automatically assign EODs to two fish. As expected, this automatic procedure was not free from errors. However, by reanalyzing a well-defined subset of the data (frames where the difference in the estimation error of two individuals was low), the precision used for identifying EOD-emitting fish was significantly improved without requiring substantial user interference (Figure 7). Based on the automated EOD-labeling and tracking of individuals, we were then able to model the sensory input that each fish experiences by applying a BEM.

The obtained data revealed that in agonistic interactions between fish of different sizes, the attack initiation appears to be mediated by electrosensory information of the contender's direction (Figure 9). Specifically, the fish that finds the electric field generated by the other fish first will initiate the approach that leads to the first contact. However, despite the finding that larger fish dominate in agonistic encounters (Figure 8), we found no clear relation of the probability to lead the attack with respect to the size difference between dyads. This makes it plausible that the RHP of the contender is not available from either passive or active images during first encounters.

Taken together, the supervised learning methods and the workflow established now should prove valuable for future studies of electrocommunication in weakly electric fish.

### Supervised Learning Algorithms to Study Interactions of Weakly Electric Fish

In this study, we used SLEAP to track individual fish from low-resolution video recordings. Compared to DeepLabCut, another open-source environment, which is capable of tracking multiple individuals (Mathis et al., 2018; Nath et al., 2019), our choice was based on the comparative ease to install SLEAP on local PCs and the stability of the GUI on our PC (Windows 10, processor: Intel®Xeon®CPU E3-1270 v5 @ 3.60GHz 3.60 GHz, working memory: 32GB RAM). However, our approach could also be implemented in DeepLabCut or similar constantly evolving tracking toolboxes (Lauer et al., 2021). To enhance the efficiency of model training and tracking, we decided to move to Colab. As the data storage size is limited in Colab, video material should not be too big and we would recommend to rely on local high-performance clusters of PCs if possible.

The EOD allocation to fish-ID was implemented based on an RFR model within the Scikit-learn package implemented in Python (Pedregosa et al., 2011). This is just one of many supervised learning methods. As an example, we tested an MLP model on the single fish recording of data set I (Figure 1). The RFR model outperformed the MLP models even for hidden layer sizes of 200 (median estimation error: RFR model: 1.48 cm; MLP: 2.18 cm). Nevertheless, both approaches were comparable with respect to their accuracy.

Our workflow was based on a laboratory condition in which visual tracking is easy. We thus used actual fish recordings for the training of the RFR model. Using real fish data instead of simulated data has the advantage that we did not have to model the boundary effects. However, more refined electric field modeling (e.g., Comsol-based simulations) could also be used. We would envision, however, that our measurement-driven approach will be swifter to implement, particularly for more complex setups than the comparatively reduced arena used here.

Once trained, the RFR model could also be used to predict the fish location without the need for additional video tracking if precision must not be very high. The median error of the model-tracked head position was about 1.48 cm, which is comparable to the data reported for other electrical fish tracking algorithms (Jun et al., 2013; average accuracy: 2.5 cm for eight-channel configuration). A more refined electrode array would further improve the precision attainable. Jun et al. (2013) used a dipole model to create a lookup table that was later used to localize fish. A dipole model can be used as a suitable approximation of the electric field of the weakly electric fish in simple environments but performance will degrade in a more complex environment as model performance is reduced near objects (Jun et al., 2013). Our approach is less sensitive to the complexity of the environment as long as the complexity is already introduced during the training sessions.

The tracking precision of the attack data set was reduced as compared to the single fish tracking data (single fish: 1.48 cm and dyadic interaction: 3.2 cm). This reduction in performance likely has several causes: the trajectories may have covered different parts of the experimental setup compared to the training data set; secondly, fish in the attack group were more heterogeneous in size than the pair used in training, and finally, the pose of animals during the dyadic interactions might have differed from the training conditions. Most likely, the different spatial coverage had the most significant effect. Even though we had split the data for the initial training into training and a test set, both still were taken from the same trajectories. Thus, the spatial coverage along these trajectories was higher than that over the remaining area of the setup. We did not attempt to quantify the magnitude of this effect. One possibility to overcome it in the future is to sample the training data set to achieve equal spatial sampling of the arena. Furthermore, similar to the retraining approach already applied for the pose estimation tracking using SLEAP, it might be useful to add the corrected EOD assignments to the training data set. Another option that would improve localization performance would be to substantially enlarge the data set used to train the model.

In principle, the training of the RFR model could also be performed with the simulated data. For this, we would recommend more sophisticated methods that incorporate a boundary effect. Alternatively, one could exclude the data obtained close to walls or close to electrodes (Jun et al., 2013). Because the fields in our case can be viewed as static and no capacitive properties needed to be included, the prior solution could be achieved using analytically methods like the finite element method or the boundary element method as used in this case to model the EIs (Gómez-Sena et al., 2014). With the improvement (even sampling during training) as mentioned earlier, we consider the training of the model with real fish data as a suitable and fast approach, as demonstrated here. For laboratory work conducted in large tanks, using an RFR model might also be a promising approach. However, it seems advisable in such a scenario to use the modeled and calibrated data as an input for training, as equal spatial coverage of larger arenas would require long sampling periods. For fieldwork, it appears questionable whether reasonably small localization errors can be achieved. At present, there are few methods available to track weakly electric fish in the field. One particularly interesting study in this regard was to estimate the position and orientation of fish (up to three animals at a time) by solving an inverse problem based on the known sensor geometry and an electrostatic dipole model through Bayesian interference (Madhav et al., 2018). The spatial precision is not sufficient to enable the resolution of directly interacting fish, but certainly the approach will be of great interest for investigating locomotor behavior on larger scales, such as territorial or foraging movements and excursions in the field.

Although we now evaluated and studied the suitability of the proposed methods and workflow for dyadic interactions, no major constraints for working with more animals exist. The RFR model estimates fish position based on the electric potentials at the electrode. The number of fish in the tank does have an influence on the precision of that measurement as they usually have a different conductivity as the water. However, as the position estimation of the RFR model is based on several electrodes, it is likely relatively robust against this interference. However, the likelihood of fish being close together and of similar or identical orientation also increases with the number of fish in the setup. Thus, the fraction of frames that will require manual inspection is expected to scale up with the number of individuals. A partial remedy to this would be a denser coverage of the arena by using more electrodes. This will be of particular relevance for setups with more water depth.

In conclusion, we are confident that our approach is also suitable to track small groups of animals. However, the limitation on the number of animals being tracked together will need to be tested for each experimental setup.

### The Role of the EI During Dyadic Interactions

Agonistic behavior is one of a variety of behaviors that are considered to be important in conflict resolution between members of the same species (Lorenz, 1963; King, 1973). It can include the emergence of individual aggression that often occurs during the formation of hierarchical relationships within populations (Kudryavtseva, 2000). The rank within a group itself is often directly related to the access to resources, including territories, sexual partners, or food. Studying social interaction that shapes this access, in this case, agonistic encounters, in electric fish is particularly advantageous: Their social behavior includes both electric and locomotor displays that in part depend on a well-known and an experimentally accessible neural circuit. Furthermore, the ability to computationally reconstruct the electrosensory information that contenders could obtain in (or prior to) agonistic encounters can provide access to a deeper understanding of behavioral choices (and their outcomes) in social interactions.

As expected from previous work with the same species as the one investigated in our work, fish length was a good predictor of the outcome of an aggressive encounter (Terleph, 2004). The larger fish always dominated the encounter by showing a higher number of attacks (Figure 8). However, when the size difference between the two fish was smaller than <20%, the dominance status could not be predicted by size alone. Furthermore, in these encounters, the aggression level was higher as revealed by the more frequent attacks (determined for 5 min after the first encounter). While size difference was predictive of the outcome of the agonistic interaction, it did not correlate with the decision which fish initiated the first approach. This may be explained in two ways: either fish cannot infer the relative size difference based on the active or passive electrosensory information received, or the size difference is ignored in the decision to attack. Meanwhile, more research is required to decide this question, our analysis of the electrosensory input allowed us to conclude that the passive electrosensory information rather than the active electrosensory information would be the source most likely to be of importance in this behavior. We found that indeed the magnitude of the passive electrosensory image perceived by the approaching fish was a suitable predictor of attack initiation. This suggests that, while passive electrolocation mediates the information about the position of a contender, the RHP (size) is either ignored or not perceived at this stage. The finding that EIs, both passive and active, were mainly focused on the head in part is explained through the anatomy rather than the trajectories. As a consequence of the tapering off of the body thickness toward the tail, which reduces the cross-sectional area and thereby increases the internal resistivity, current (generated either by the fish itself or by external sources) is funneled to the head region. As a result, the maximal current densities (and transcutaneous voltages) occur at the head region. This could aid in the detection of contenders and the assessment of their RHP. While the latter was not found in our study, the finding that passive EIs likely provide (spatial) information to the fish initiating the first contact agrees well with the results of dyadic interactions in *G. omarorum*, a South American weakly electric fish species (Pedraja et al., 2016). The similarity suggest that the role of the EI in agonistic encounters may be shared between different independently evolved electric fish species. With respect to the methodological aspect, the methods established now seem suitable to further our understanding of the role of active and passive electroreception in different situations: agonistic contest, courtship display, exploration of objects, and determination of different perceptual parameters in collective behavior.

## Data Availability Statement

The raw data supporting the conclusions of this article will be made available by the authors, without undue reservation.

## Ethics Statement

The animal facility and housing conditions are in accordance with European and federal regulations. The behavioral observations were conducted as evaluated and approved by the corresponding federal agency (LANUV, 84-02.04.2017.A151).

## Author Contributions

SJ and JE designed the experiments. SJ conducted the behavioral experiments. SJ, JE, and FP wrote the manuscript. FP modeled the electric images during the dyadic interactions. HH analyzed the dipol model and designed the Random Forest Network to test for optimal setup configurations. All authors contributed to the article and approved the submitted version.

## Funding

Part of this study was supported by the DFG (EN 826/8-1).

## Conflict of Interest

The authors declare that the research was conducted in the absence of any commercial or financial relationships that could be construed as a potential conflict of interest.

## Publisher's Note

All claims expressed in this article are solely those of the authors and do not necessarily represent those of their affiliated organizations, or those of the publisher, the editors and the reviewers. Any product that may be evaluated in this article, or claim that may be made by its manufacturer, is not guaranteed or endorsed by the publisher.
